# Chronic Constrictive Pericarditis

**DOI:** 10.1155/2013/957497

**Published:** 2013-09-24

**Authors:** Hossein Doustkami, Afshin Hooshyar, Nasrollah Maleki, Zahra Tavosi, Iraj Feizi

**Affiliations:** ^1^Interventional Cardiology, Department of Cardiology, Imam Khomeini Hospital, Ardabil University of Medical Sciences, Iran; ^2^Department of Internal Medicine, Imam Khomeini Hospital, Ardabil University of Medical Sciences, Ardabil, Iran; ^3^Department of Internal Medicine, Shohadaye Khalije Fars Hospital, Bushehr University of Medical Sciences, Bushehr, Iran; ^4^Department of Cardiothoracic Surgery, Imam Khomeini Hospital, Ardabil University of Medical Sciences, Ardabil, Iran

## Abstract

Constrictive pericarditis (CP) is a rare clinical entity that can pose diagnostic problems. The diagnosis of CP requires a high degree of clinical suspicion. The gold standard for diagnosis is cardiac catheterization with analysis of intracavitary pressure curves, which are high and, in end diastole, equal in all chambers. We present a patient with unexplained dyspnea, recurrent right-side pleural effusion, and ascites. Analysis of the ascitic fluid revealed a high protein content and an elevated serum-ascites gradient. Echocardiography, computed tomography, and cardiac catheterization revealed the diagnosis of CP. He underwent complete pericardiectomy and to date has made a good recovery. The diagnosis of CP is often neglected by admitting physicians, who usually attribute the symptoms to another disease process. This case exemplifies the difficulty in diagnosing this condition, as well as the investigation required, and provides a discussion of the benefit and outcomes of prompt treatment.

## 1. Introduction

Constrictive pericarditis (CP) is a disease characterized by the encasement of the heart by a rigid nonpliable pericardium due to dense fibrosis and adhesions. This causes impaired diastolic cardiac function [1]. Patients with pericardial constriction may present with two types of complaints: those related to fluid overload, ranging from peripheral edema to anasarca; and those related to diminished cardiac output response to exertion, such as fatigability and dyspnea on exertion. Pericardial constriction should be considered in any patient with an unexplained elevation in jugular venous pressure, particularly if there is a history of a predisposing condition [2]. The common cause of this disease is idiopathic or viral pericarditis. Other causes include tuberculosis, trauma, cardiac surgery, irradiation with mediastinum, septic infections, histoplasmosis, systemic lupus erythematosus, rheumatoid arthritis, malignancies, and chronic kidney disease along with chronic dialysis [2–5]. Pericardial disease rarely presents as the initial manifestation tuberculosis [6–9]. Cardiac CT and MRI can detect pericardial thickening and calcification with high accuracy [10]. Echocardiography is very useful for differential diagnosis between CP and restrictive cardiomyopathy [11, 12]. The gold standard for diagnosis is cardiac catheterization. Pericardiectomy is the only definitive treatment of CP and should be as complete as possible [4, 13, 14].

## 2. Case Report

**Figure 1:**
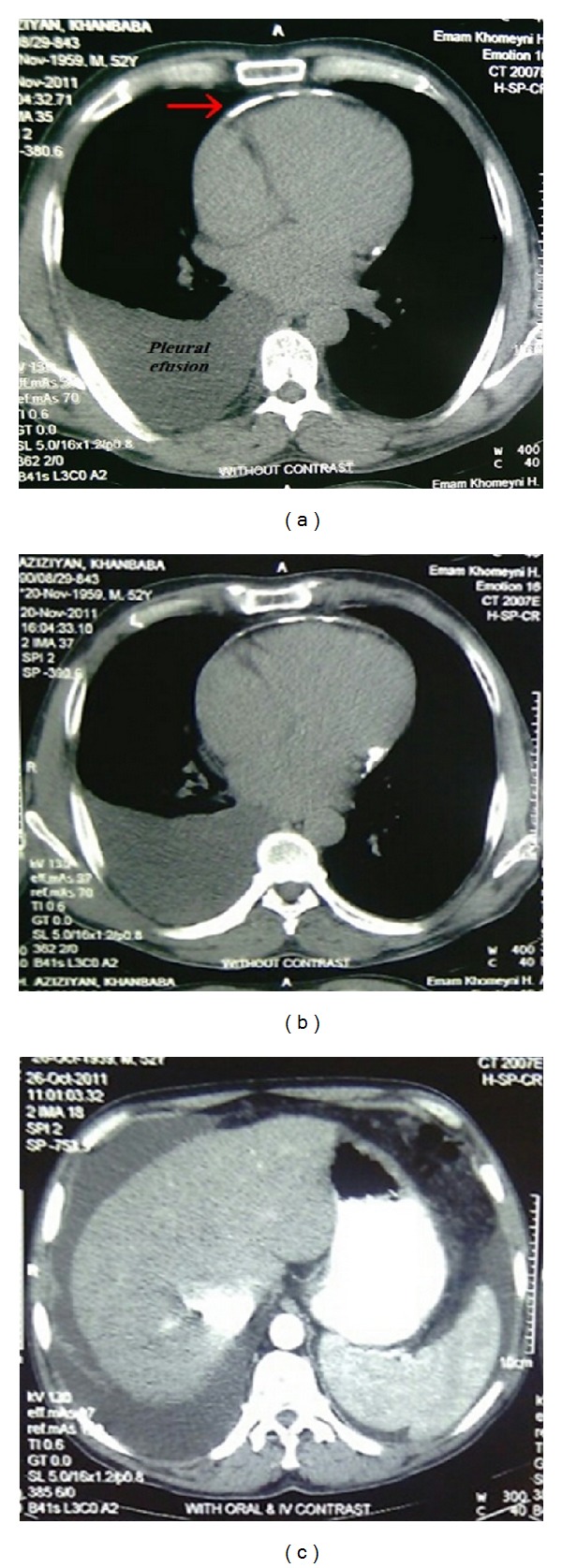
Chest CT showing pleural effusion, cardiac calcification, ascites, and IVC dilation.

**Figure 2:**
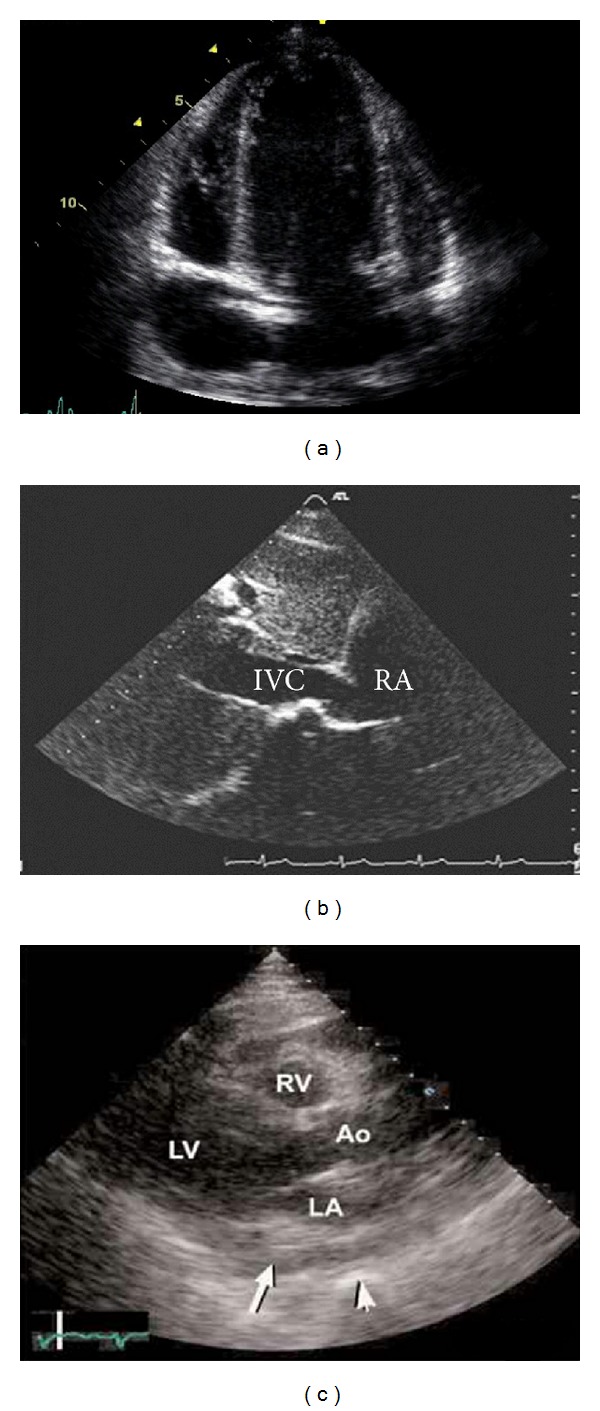
Echocardiography showing septal bouncing (a), dilation of IVC (b), pericardial effusion, and calcification (c).

The patient is a 52-year-old man who gradually suffered since about 5 years from exertional dyspnea, weakness and lack of energy, fatigue feeling, pleuritic chest pain, distension of abdomen, and peripheral edema. Patient has a past history of hospitalization one year ago due to chest pain and received coronary angiography, and it was normal. The patient also received diagnostic thoracentesis 6 months before due to dyspnea and the presence of right-side pleural effusion, and he had exudative pleural effusion with lymphocyte-dominant and nondiagnostic cytology, and for this reason, he received thoracoscopy and pleural biopsy which were nondiagnostic. The patient referred to our hospital due to pain and progressive abdominal distention in the past 10 days was hospitalized. On physical examination, the patient was hemodynamically stable (blood pressure was 110/80 mmHg and pulse was 78 beats per minute). JVP was very elevated. Heart sounds were muffle, and reduction of sound was found at the base of the right lung. In the examination, mild hepatomegaly with ascites and peripheral edema was seen. Primary laboratory evaluations were normal. Analysis of the ascitic fluid revealed a high protein content (4.1 g/dL) and an elevated serum-ascites gradient (1.6 g/dL). In abdominal sonography, congestive hepatomegaly, mild splenomegaly, ascites, and evidence of portal hypertension were seen. In upper endoscopy, esophageal varices were not seen and viral hepatitis serology was negative. In chest and abdominal CT, right pleural effusion, pericardial thickness and calcification, ascites, and inferior vena cava dilation were seen (Figure 1). To study abdominal vascular thrombosis, MRV (Magnetic Resonance Venography) was performed, and the results were normal. In the conducted echocardiography, enlargement of right atrium (44 mm), right ventricle (46 mm), and left atrium (42 mm) along with mild pericardial effusion, pericardial calcification, inferior vena cava dilation (28 mm) and septal bouncing was found (Figure 2). 

**Figure 3:**
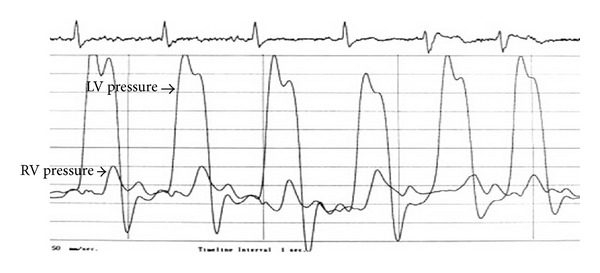
The equalization of diastolic pressures and “square root sign” or “dip and plateau sign” of the left ventricular waveforms.

Right and left cardiac catheterization were performed for the patient in which elevation and equalization of right atrial pressure (29 mmHg), pulmonary capillary wedge pressure (30 mmHg), mean pulmonary arterial pressure (33 mmHg), right ventricular diastolic pressure (30 mmHg), and left ventricular diastolic pressure (30 mmHg) were found. Curves recorded in right heart catheterization indicate increase of superior vena cava pressure at time of breath and descending, and then horizontal curve of right ventricular pressure (square root sign) was evident (Figure 3). Coronary angiography was normal. All of the findings were consistent with CP. 

**Figure 4:**
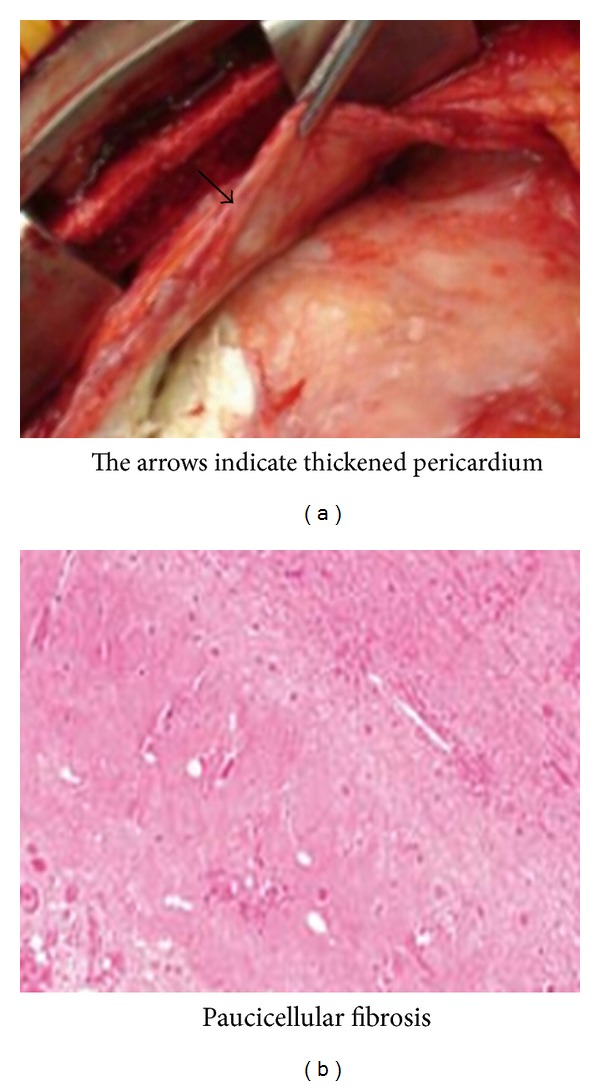
Surgical and pathological findings.

The patient underwent cardiac surgery during which pericardium was fully thick and calcified (Figure 4(a)), and received pericardiectomy. Pathological study of pericardial sample indicated fibrous pericarditis without granuloma (Figure 4(b)). Gram's staining, staining for acid-fast bacilii, and culture of the pericardium for bacteria, fungus and acid-fast bacilli were negative. 

One year after the operation, the patient reported a dramatic improvement in his exertion tolerance, along with decrease of dyspnea and distension of abdomen. 

## 3. Discussion and Conclusion

This case illustrates an unusual cause of ascites. The most common cause of ascites in the United States is cirrhosis, followed distantly by cancer, right-sided heart failure, tuberculosis, pancreatic disease, and various rare infection and hematologic diseases [15]. A serum-ascites albumin gradient ≥1.1 g/dL and an ascites fluid total protein >2.5 g/dL are typical of CP and other postsinusoidal causes of ascites. Sinusoidal diseases, such as liver cirrhosis, exhibit a serum-ascites albumin gradient >1.1 g/dL but an ascites fluid total protein <2.5 g/dL [16]. When ascites is present, estimation of the jugular venous pressure is critical, since it can frequently separate cardiac from noncardiac causes. Elevated jugular venous pressure can be challenging to detect, even when the assessment is made by experienced clinicians. The overall correlation between clinical assessment of the jugular venous pressure direct measurement of central venous pressure by central venous catheterization is poor; an overall accuracy of 56% has been reported in classifying the central venous pressure as low, normal, or high, with a sensitivity for detection of a high central venous pressure (>10 cm of water) of less than 60% [17–19]. In this case, the failure to recognize the elevated jugular venous pressure led to a delay in diagnosis and extensive diagnostic testing. Symptoms of CP are typically related to systemic venous congestion and low cardiac output. Whereas elevated jugular venous pressure was present in nearly all patients with CP in a large case series, peripheral edema was absent in approximately 25% of patients, particularly early in the disease process, and less than 6% of patients presented with predominantly abdominal symptoms [2]. Therefore, a high index of suspicion is required to diagnose this entity, especially in patients with elevated protein-count ascites, jugular venous distention, and no cardiopulmonary symptoms. Pleural effusion occurs in 44–50% of patients with CP [4, 20]. Tomaselli and coworkers retrospectively analyzed 30 patients who presented with CP and found that 60% (18 patients) had pleural effusion [21]. Bilateral and symmetrical effusions were found in 12 patients, and the remaining 6 had unilateral pleural fluid (3 had right-side effusion and 3 had left-side effusion). Our patient had left side pleural effusion. Pericardial thickening detected on CT or MRI is absent in up to 28% of patients with surgically proven CP [13]. Our patient had right side heart failure and a typical cardiac CT calcification. Typical echocardiographic findings, such as normal systolic function, a plethoric inferior vena cava, a restrictive mitral inflow pattern with respiratory variation, reversal of expiratory hepatic vein flow, a septal motion suggestive of enhanced ventricular interaction, or an elevated early diastolic mitral annular velocity (*E*′) detected by tissue Doppler imaging, may not be observed if images are poor or if CP is not explicitly noted as a potential diagnosis [22, 23]. Elevated and equalized diastolic pressures on cardiac catheterization are the rule for CP. Ventricular filling is rapid early and blunted late by the stiffened pericardial sac, leading to the characteristic steep y descent of right atrial pressure and the dip and plateau of ventricular pressure [24, 25]. Although these hemodynamic patterns can be observed in other causes of heart failure such as restrictive cardiomyopathy, discordance between changes in right and left ventricular systolic pressures during respiration, known as ventricular interdependence, reliably distinguishes CP from these other conditions [13, 26]. Most patients with CP required surgical pericardiectomy. Removal of densely adherent pericardium is usually successful but can be extremely challenging [2]. Moreover, recovery can be delayed for several weeks, and patients in whom the constriction has progressed to the point of abnormal ventricular function, severely reduced cardiac output, cachexia, or end-organ dysfunction derive the least benefit from the procedure [4, 27], an observation that under-scores the importance of prompt diagnosis and treatment.

The diagnosis of CP in our patient was probably delayed from two reasons: the rarity of the diagnosis and the failure to recognize the elevated jugular venous pressure on initial examination. This case reminds us that reconsideration of clinical information from a different angel can facilitate the diagnostic process in patients with complex conditions. In conclusion, in case there is any calcification in a cardiac CT with right-sided heart failure symptoms, we should consider the diagnosis of constrictive pericarditis and performing further cardiac investigations.

## References

[1] Myers RBH, Spodick DH (1999). Constrictive pericarditis: clinical and pathophysiologic characteristics. *American Heart Journal*.

[2] Ling LH, Oh JK, Schaff HV (1999). Constrictive pericarditis in the modern era: evolving clinical spectrum and impact on outcome after pericardiectomy. *Circulation*.

[3] Cameron J, Oesterle SN, Baldwin JC, Hancock EW (1987). The etiologic spectrum of constrictive pericarditis. *American Heart Journal*.

[4] Bertog SC, Thambidorai SK, Parakh K (2004). Constrictive pericarditis: etiology and cause-specific survival after pericardiectomy. *Journal of the American College of Cardiology*.

[5] Sengupta PP, Eleid MF, Khandheria BK (2008). Constrictive pericarditis. *Circulation Journal*.

[6] Bashi VV, John S, Ravikumar E, Jairaj PS, Shyamsunder K, Krishnaswami S (1988). Early and late results of pericardiectomy in 118 cases of constrictuve pericarditis. *Thorax*.

[7] Pedreira Pérez M, Virgós Lamela A, Crespo Mancebo FJ, Cervantes JL, Fernández de la Reguera G, Barragán García R (1987). 40 years’ experience in the surgical treatment of constrictive pericarditis. *Archivos del Instituto de Cardiologia de Mexico*.

[8] Raffa H, Mosieri J (1990). Constrictive pericarditis in Saudi Arabia. *East African Medical Journal*.

[9] Arsan S, Mercan S, Sarigul A (1994). Long-term experience with pericardiectomy: analysis of 105 consecutive patients. *Thoracic and Cardiovascular Surgeon*.

[10] Rienmuller R, Gurgan M, Erdmann E, Kemkes BM, Kreutzer E, Weinhold C (1993). CT and MR evaluation of pericardial constriction: a new diagnostic and therapeutic concept. *Journal of Thoracic Imaging*.

[11] Maisch B, Seferović PM, Ristić AD (2004). Guidelines on the diagnosis and management of pericardial diseases: executive summary. The Task Force on the Diagnosis and Management of Pericardial Diseases of the European Society of Cardiology. *European Heart Journal*.

[12] Ha J-W, Ommen SR, Tajik AJ (2004). Differentiation of constrictive pericarditis from restrictive cardiomyopathy using mitral annular velocity by tissue Doppler echocardiography. *American Journal of Cardiology*.

[13] Talreja DR, Edwards WD, Danielson GK (2003). Constrictive pericarditis in 26 patients with histologically normal pericardial thickness. *Circulation*.

[14] Chowdhury UK, Subramaniam GK, Kumar AS (2006). Pericardiectomy for constrictive pericarditis: a clinical, echocardiographic, and hemodynamic evaluation of two surgical techniques. *Annals of Thoracic Surgery*.

[15] Runyon BA (1994). Current concepts: care of patients with ascites. *The New England Journal of Medicine*.

[16] Howard JP, Jones D, Mills P, Marley R, Wragg A (2012). Recurrent ascites due to constrictive pericarditis. *Frontline Gastroenterology*.

[17] Cook DJ (1990). Clinical assessment of central venous pressure in the critically ill. *American Journal of the Medical Sciences*.

[18] Cook DJ, Simel DL (1996). Does this patient have abnormal central venous pressure?. *Journal of the American Medical Association*.

[19] Connors AF, McCaffree DR, Gray BA (1983). Evaluation of right-heart catheterization in the critically ill patient without acute myocardial infarction. *The New England Journal of Medicine*.

[20] Wychulis AR, Connolly DC, McGoon DC (1971). Surgical treatment of pericarditis. *Journal of Thoracic and Cardiovascular Surgery*.

[21] Tomaselli G, Gamsu G, Stulbarg MS (1989). Constrictive pericarditis presenting as pleural effusion of unknown origin. *Archives of Internal Medicine*.

[22] Oh JK, Hatle LK, Seward JB (1994). Diagnostic role of Doppler echocardiography in constrictive pericarditis. *Journal of the American College of Cardiology*.

[23] Troughton RW, Asher CR, Klein AL (2004). Pericarditis. *The Lancet*.

[24] Hansen AT, Eskildsen P, Gotzsche H (1951). Pressure curves from the right auricle and the right ventricle in chronic constrictive pericarditis. *Circulation*.

[25] Meaney E, Shabetai R, Bhargava V (1976). Cardiac amyloidosis, constrictive pericarditis and restrictive cardiomyopathy. *American Journal of Cardiology*.

[26] Hurrell DG, Nishimura RA, Higano ST (1996). Value of dynamic respiratory changes in left and right ventricular pressures for the diagnosis of constrictive pericarditis. *Circulation*.

[27] Ha J-W, Oh JK, Schaff HV (2008). Impact of left ventricular function on immediate and long-term outcomes after pericardiectomy in constrictive pericarditis. *Journal of Thoracic and Cardiovascular Surgery*.

